# Oligotrophic bacterium *Hymenobacter latericoloratus* CGMCC 16346 degrades the neonicotinoid imidacloprid in surface water

**DOI:** 10.1186/s13568-019-0942-y

**Published:** 2020-01-14

**Authors:** Leilei Guo, Zhiling Dai, Jingjing Guo, Wenlong Yang, Feng Ge, Yijun Dai

**Affiliations:** 1 Jiangsu Key Laboratory for Microbes and Functional Genomics, Jiangsu Engineering and Technology Research Center for Industrialization of Microbial Resources, College of Life Science, Nanjing Normal University, Nanjing, 210023 People’s Republic of China; 2 Jiangsu Collaborative Innovation Center of Atmospheric Environment and Equipment Technology, Nanjing University of Information Science & Technology, Nanjing, 210044 People’s Republic of China

**Keywords:** Degradation, Hydroxylation, Imidacloprid, *Hymenobacter latericoloratus*, Clusters of Orthologous Groups

## Abstract

The intensive and extensive application of imidacloprid in agriculture has resulted in water pollution and risks to aquatic invertebrates. However, pure bacteria remediation of imidacloprid in surface water environments has not been studied. Here, we isolated an imidacloprid-degrading bacterium from a water environment, examined its imidacloprid degradation in pure culture and surface water, sequenced its genome, and compared its Clusters of Orthologous Groups (COG) protein categorization with that for another imidacloprid-degrading bacterium. The isolate was an obligate oligotrophic bacterium, *Hymenobacter latericoloratus* CGMCC 16346, which degraded imidacloprid via hydroxylation by co-metabolism in pure culture. Resting cells degraded 64.4% of 100 mg/L imidacloprid in 6 days in the presence of co-substrate maltose, and growing culture degraded 40.8% of imidacloprid in 10 days. *H. latericoloratus* CGMCC 16346 degraded imidacloprid in surface water without co-substrate supplementation and retained imidacloprid-degrading activity after 30 days. The half-life of imidacloprid in surface water was decreased from 173.3 days in the control to 57.8 days by CGMCC 16346 inoculation. Genome sequencing and COG analysis indicated that carbohydrate metabolism and transport, cell wall/membrane biogenesis, and defense mechanisms are enriched in *H. latericoloratus* CGMCC 16346 compared with the copiotrophic imidacloprid-degrading *Pseudoxanthomonas indica* CGMCC 6648, indicating that *H. latericoloratus* CGMCC 16346 is adapted to live in oligotrophic water environments and biofilms. *H. latericoloratus* CGMCC 16346 is a promising bioremediation agent for elimination of imidacloprid contamination from surface water.

## Introduction

Imidacloprid (*N*-{1-[(6-chloro-3-pyridyl)methyl]-4,5-dihydroimidazol-2-yl}nitramide) is a systemic neonicotinoid insecticide that acts on the central nervous system of pest insects. It is one of the most widely used insecticides in the world, to control insects including aphids, leafhoppers, planthoppers, thrips, termites and whiteflies. As a potent neurotoxin-type insecticide that is often applied to crops as soil drench, foliar spray and seed treatment, imidacloprid has been implicated in a variety of ecosystem effects, particularly declines in populations of both wild and domestic bees (Eng et al. 2017). Because imidacloprid and the other neonicotinoids clothianidin and thiamethoxam pose an unacceptably high risk to bees, the European Union decided in April 2018 to ban these neonicotinoids for all outdoor uses. Nevertheless, imidacloprid is still widely applied in North America and Australia, and because it is extremely effective against many hemipteran insect pests, it has been extensively used in rice-planting regions of Asia to control rice planthoppers (Sánchez-Bayo and Hyne 2014; Bradford et al. 2018).

Imidacloprid persists in soils for a year or more and is highly mobile in soil, eventually moving into surface waters or leaching into groundwater. Recent surveys of imidacloprid detection from the USA, Netherlands, Australia, Sweden, Vietnam and China have confirmed water contamination by imidacloprid (Lamers et al. 2011; van Dijk et al. 2013; Morrissey et al. 2015). In these surveys, imidacloprid residues were detected in 78–100% of cases in surface waters at concentrations often exceeding the benchmarks for protection of aquatic organisms in the respective countries. Typically, imidacloprid residue levels in surface waters are below 1 μg/L, with maximum concentrations for imidacloprid ranging from 0.22 μg/L in Vietnam to 25 μg/L in USA, but can reach as high as 200 μg/L in the Netherlands (van Dijk et al. 2013). Recently, Klarich et al. (2017) found the presence of imidacloprid in finished drinking water, demonstrating its persistence during conventional water treatment.

Global imidacloprid contamination of the water system has caused public concern and researchers have tried to find a simple method to remove imidacloprid from surface and groundwater, and to eliminate it from contaminated aqueous effluents. Socíasviciana et al. (2003) developed the removal of imidacloprid from water by heat-treated kerolites. Redlich et al. (2007) investigated the photochemical degradation of imidacloprid. Tang et al. (2012) performed photoinduced degradation of imidacloprid in aqueous solutions in the presence of TiO_2_ as photocatalyst. Klarich et al. (2017) used granular activated carbon filtration to lower the concentration of imidacloprid in finished water from an Iowa City treatment facility.

**Fig. 1:**
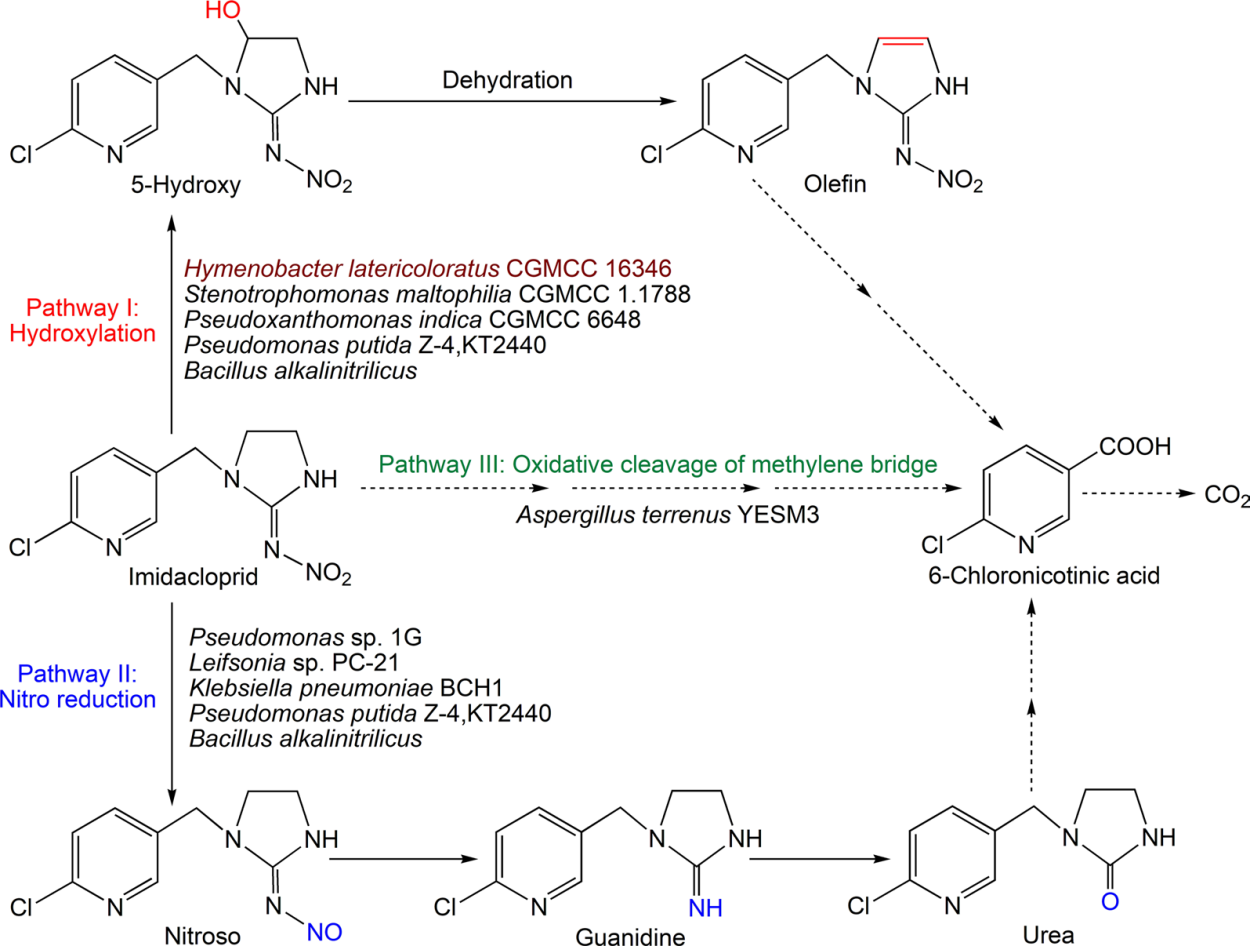
Metabolic pathway of imidacloprid in microorganisms

Microbial degradation is a clean, efficient and ecofriendly approach to remediation of organic compounds in soil and water. Several bacteria isolated from soil, such as *Bacillus alkalinitrilicus* (Sharma et al. 2014), *Klebsiella pneumoniae* BCH1 (Phugare et al. 2013), *Leifsonia* sp. PC-21 (Anhalt et al. 2007), *Mycobacterium* sp. MK6 (Kandil et al. 2015), *Pseudomonas* sp. 1G (Pandey et al. 2009), *Pseudomonas* sp. RPT 52 (Gupta et al. 2016), *Pseudomonas putida* KT2440 and Z-4 (Lu et al. 2016), *Pseudoxanthomonas indica* CGMCC 6648 (Ma et al. 2014), and *Stenotrophomonas maltophilia* CGMCC 1.1788 (Dai et al. 2006), and the fungus *Aspergillus terreus* YESM3 (Mohammed and Badawy 2017) isolated from waste water, have been reported to degrade imidacloprid in pure culture. The metabolic pathways of imidacloprid degradation by these microbes are shown in Fig. 1. However, microbial degradation and remediation of imidacloprid in water systems has not been studied. The fate of imidacloprid in aquatic systems indicates that it undergoes degradation via photolytic reactions or microbial activity. Although imidacloprid undergoes photolysis quickly, it remains in the water column in aquatic systems, and has an aerobic sediment and water half-lifetime of 30 to 162 days (Bonmatin et al. 2015). Research suggests that imidacloprid is generally persistent in water and not easily biodegradable (van Dijk et al. 2013; Lu et al. 2016). Therefore, it is important to screen and isolate microbes with the ability to degrade imidacloprid in water.

In the isolation of pesticide-degrading microbes, nutrient medium (as broth or in an agar plate) is generally used to purify and cultivate microbes that grow on mineral medium supplemented with organic pesticide as the sole nitrogen source or carbon and energy source. Therefore, copiotrophic bacteria that easily grow on nutrient medium are more readily isolated than oligotrophic bacteria, and obligate oligotrophic bacteria that cannot grow in nutrient medium may be inadvertently ignored. Natural surface waters usually contain low levels of dissolved organic and inorganic nutrients (Yang et al. 2007) and only oligotrophic bacteria are able to live in these conditions (Xia and Liang 2006). This makes oligotrophic bacteria suitable for bioremediation of low-level organic pesticide contamination in natural surface water with low levels of nutrients.

In the present study, we focused on the isolation of oligotrophic bacteria from water samples, and tested their ability to degrade imidacloprid in pure culture and surface water in laboratory conditions. An imidacloprid-degrading isolate was obtained, its genome was sequenced, and Clusters of Orthologous Groups (COG) categories of its predicted proteins were compared with those of the copiotrophic bacterium *Pseudoxanthomonas indica* CGMCC 6648, an imidacloprid-degrading bacterium isolated from soil (Ma et al. 2014). Our studies will help to reduce imidacloprid contamination in water environments and the genome annotation and COG comparison will help with understanding the oligotrophic lifestyle of microbes, as well as being useful in developing strategies to screen microbes for remediation of water contamination.

## Materials and methods

### Chemicals

Imidacloprid was provided by Jiangsu Pesticide Research Institute Company Ltd., Nanjing, China (98% purity). 5-Hydroxy imidacloprid was synthesized according to the methods described in our previous report (Dai et al. 2007). Other reagents were of analytical grade and purchased from commercial agents, except acetonitrile was of high-performance liquid chromatography (HPLC) grade and purchased from Tedia Co. Ltd. (Fairfield, OH, USA).

### Strains and media

Mineral salt medium (MSM; pH 7.0) contained 2.1 g Na_2_HPO_4_, 1.4 g KH_2_PO_4_, 0.5 g MgSO_4_·7H_2_O, and 10 mL metal ion solution in 1 L deionized water. The metal ion solution contained 0.1 g KI, 0.3 g H_3_BO_3_, 0.4 g CaCl_2_·2H_2_O, 0.04 g CuSO_4_·5H_2_O, 0.2 g FeSO_4_·7H_2_O, 0.4 g MnSO_4_·7H_2_O, 0.2 g NaMoO_4_·2H_2_O, and 1% concentrated hydrochloric acid in 1 L deionized water. MSM supplemented with 100 mg/L imidacloprid was used for enrichment of imidacloprid-degrading microbes. The low-nutrient Reasoner’s 2A (R2A) medium (pH 7.0) containing 0.25 g tryptone, 0.25 g peptone, 0.5 g casein acid hydrolysate, 0.5 g soluble starch, 0.5 g glucose, 0.3 g sodium pyruvate, 0.1 g MgSO_4_·7H_2_O, and 0.3 g K_2_HPO_4_ in 1 L deionized water was used for bacterial isolation and cell culture. The oligotrophic nutrient medium was 1000- and 10,000-fold diluted lysogeny broth (LB) (pH 7.2); 100% LB contained 10 g tryptone, 5 g yeast extract and 10 g NaCl in 1 L deionized water. Solid medium included 2% agar.

### Isolation and identification of an imidacloprid-degrading bacterium

Water samples and water–sediment were collected from a lake in the Xianlin campus of Nanjing Normal University, Nanjing, Jiangsu Province; Ganzhou City, Jiangxi Province; Heze City, Shandong Province; and Shangqiu City, Henan Province (all in China). The water samples were spread directly on MSM agar plates. The water-sediments were diluted tenfold and mixed through vortex oscillation for 5 min. Then, 0.1 mL supernatant was spread on an MSM agar plate. The plates were incubated at 30 °C until single colonies appeared. The single colonies were streaked onto R2A agar plates for purification and incubated at 30 °C.

To simply and rapidly screen imidacloprid-degrading microbes, each isolate was streaked on an R2A plate with a single line (about 2 cm long). After growth, every colony was scraped and suspended in a 50-mL sterilized plastic centrifuge tube containing 2 mL of 50 mmol/L sterilized phosphate buffer (pH 7.0). Imidacloprid (100 mg/L) and glucose (20 g/L) were added by filter sterilization. The centrifuge tube was sealed with a breathable membrane and incubated on a rotary shaker at 200 rpm and 30 °C. After incubation for 4 days, the samples were centrifuged at 8000×*g* for 10 min to remove the cells and the supernatant was collected. Acetone (25% volume) was added, and the solution was filtered through a 0.22-μm pore size membrane. The filtrate was used for analysis of the substrate and metabolites by HPLC. Bacterial isolates capable of degrading imidacloprid were taxonomically identified by morphological observation and 16S rRNA gene sequence analysis. The 16S rRNA gene was amplified by colony PCR. The PCR system and conditions, agarose gel electrophoresis analysis, sequence alignment and phylogenetic tree construction were described in our previous report (Ge et al. 2014).

### Identification of bacteria as oligotrophic

R2A culture broth inoculated with an isolated bacterium and incubated for 16 h was diluted to 10^−5^ by gradient dilution and then 100 μL of the dilution was placed onto an oligotrophic nutrient medium agar plate and spread uniformly. An agar plate excluding nutrient medium was used as a negative control. The plates were incubated at 30 °C for 10 days. Bacteria that grew obvious colonies on the 1000- and 10,000-fold diluted LB agar plate were identified as oligotrophic (Han et al. 2014).

### Test of imidacloprid biodegradation by growing culture and resting cells

The imidacloprid-degrading isolate was inoculated onto R2A agar plates and incubated at 30 °C for 30 h, then a single colony was inoculated into a 100-mL flask containing 20 mL R2A broth and the flask was incubated on a rotary shaker at 200 rpm and 30 °C. After incubation for 16 h, OD_600_ reached about 2; 1 mL of this broth, defined as seed broth, was inoculated into a 500-mL flask containing 100 mL R2A broth and filter-sterilized imidacloprid (added to 100 mg/L). The growing culture broth was sampled every 24 h.

For examination of resting cell transformation of imidacloprid by the isolated bacterium, the above seed broth was inoculated into R2A broth without imidacloprid and incubated for 14 h. The cells were harvested by centrifugation at 6000×*g* for 10 min and then the cell sediments were washed with 50 mmol/L autoclaved sodium phosphate buffer (pH 7.0) and subsequently resuspended in the same buffer supplemented with 100 mg/L (final concentration) filter-sterilized imidacloprid. The cell density was OD_600_ = 5.0; 2 mL of the cell suspension was added into an autoclaved 50-mL centrifuge tube and then the centrifuge tube was sealed with a breathable membrane and incubated on a rotary shaker at 200 rpm and 30 °C. The resting cell transformation system excluding imidacloprid or cells was used as controls.

To test the effect of co-substrate on imidacloprid degradation, 20 g/L filter-sterilized co-substrate was added into resting cell transformation broth. The transformations were conducted in the above cultivation conditions. The samples were taken every 24 h and that used for HPLC analysis were prepared by the same methods as for the above growing culture transformation.

### Biodegradation of imidacloprid in surface water

Surface water was sampled from CaiYue Lake, at the Xianlin campus of Nanjing Normal University, Nanjing, China. The physicochemical properties of the surface water were: total phosphorus 0.07 mg/L, total Kjeldahl nitrogen 2.35 mg/L, and chemical oxygen demand 4.87 mg/L; pH 7.3. The samples were filtered through a 0.22-μm pore-size water-phase membrane. Imidacloprid was added into the water to 10 mg/L. Washed cells were added at 2 × 10^8^ cells/mL. Then, 20 mL of the mixture was added into a 100-mL flask. A similar sample without bacterial inoculation was used as a control. The flasks were incubated at 30 °C in a rotary shaker at 200 rpm. After incubation for 10 days, 1 mL of the sample was collected and centrifuged at 10,000×*g* for 10 min to remove bacterial cells, and the supernatant was filtered using a 0.22-μm pore-size membrane before HPLC analysis.

Imidacloprid biodegradation in surface water was scaled-up in a Sunsun HR-180 tank equipped with water circulation and aerator systems (Sensen Co., Ltd., Wuxi, China). The tested volume of surface water was 2.5 L. The initial concentration of imidacloprid in the tank was 10 mg/L. Bacterial cells were inoculated to 3 × 10^7^ cells/mL. In fed-batch bacterial inoculation, the same amount of cells was supplied on day 0, 10 and 20, respectively. Every 5 days, samples were prepared by centrifugation and filtration for HPLC analysis.

### HPLC and liquid chromatography-mass spectrometry (LC–MS) analyses

An Agilent 1200 series HPLC system and an Agilent 1290 infinity LC with a G1315B diode-array detector and an Agilent 6460 Triple Quadrupole LC–MS system were used for the quantitative analysis of imidacloprid and its metabolites and the metabolite identification. The column, mobile phase, and monitored wavelength were described in our previous report (Lu et al. 2016) The flow rate for the column elution for HPLC analysis and LC–MS analysis was 1 and 0.6 mL/min, respectively. In these conditions, the metabolites olefin imidacloprid, 5-hydroxy imidacloprid and imidacloprid appeared at retention times of 6.0, 6.9 and 9.6 min, respectively in HPLC analysis, and 9.8, 11.2 and 16.0 min, respectively in LC–MS analysis. Electrospray ionization was operated in the negative ionization mode.

### Imidacloprid half-life

Half-life periods were determined according to the method described by Suchail et al. (2004). In all cases, the first order equation provided a satisfactory fit for the data (*r*^2^ > 0.9), providing the basis for the half-life calculation.

### Genome sequencing and annotation

The complete genome sequence of the isolate was generated by BGI Tech Solutions Co., Ltd. (Shenzhen, China) using an Illumina HiSeq 4000 platform and PacBio RS II platform. Software Falcon 0.3.0, proovread 2.12, Celera Assembler 8.3, SMRT Analysis 2.3.0, and GATK 1.6–13 were used for genome assembly.

Genes were predicted using Glimmer 3.02 software. rRNAs were identified by comparing with the rRNA database or prediction with RNAmmer 1.2 software. tRNAscan-SE 1.3.1 was used to predict tRNAs and their secondary structure. sRNAs were predicted using Infernal and comparison with the Rfam database. Tandem Repeat Finder 4.0.4 software was used to predict tandem repeat sequences. Seven databases—Kyoto Encyclopedia of Genes and Genomes, COG, Non-Redundant Protein, Swiss-Prot, Gene Ontology, TrEMBL and EggNOG—were used for functional annotation.

### Piperonyl butoxide (PBO) inhibition

Inhibition of imidacloprid degradation and 5-hydroxy imidacloprid formation by PBO was tested using the method in our previous report (Dai et al. 2007). PBO was dissolved in acetone with concentration 100 mmol/L, then 10 μL of the PBO solution was added into standard resting cell transformation broth (2 mL) to give a PBO concentration of 0.5 mmol/L. To the control, 10 μL of acetone were added. After incubation for 2 days, the sample was analyzed for the concentration of imidacloprid and 5-hydroxy imidacloprid by HPLC analysis.

### Genome comparison of *H. latericoloratus* CGMCC 16346 and *P. indica* CGMCC 6648

The *H. latericoloratus* CGMCC 16346 genome (GenBank accession number of the chromosome: CP040936, GenBank accession number of the plasmid: CP040937) was compared that of *P. indica* CGMCC 6648 (GenBank accession number: GCA_006542425.1). The Cluster of Orthologous Groups of proteins (COG) of *H. latericoloratus* CGMCC 16346 and *P. indica* CGMCC 6648 was compared.

## Results

### Isolation of imidacloprid-degrading microbe and taxonomic identification

About 200 colonies grown on MSM agar were streaked onto R2A plates and eight different bacterial morphologies were observed among the growing colonies. These eight types of bacteria were examined for their ability to degrade imidacloprid by HPLC. A pink bacterium named DG01 could degrade imidacloprid and produced the metabolite 5-hydroxy imidacloprid, while none of the other strains had imidacloprid degradation activity.

**Fig. 2:**
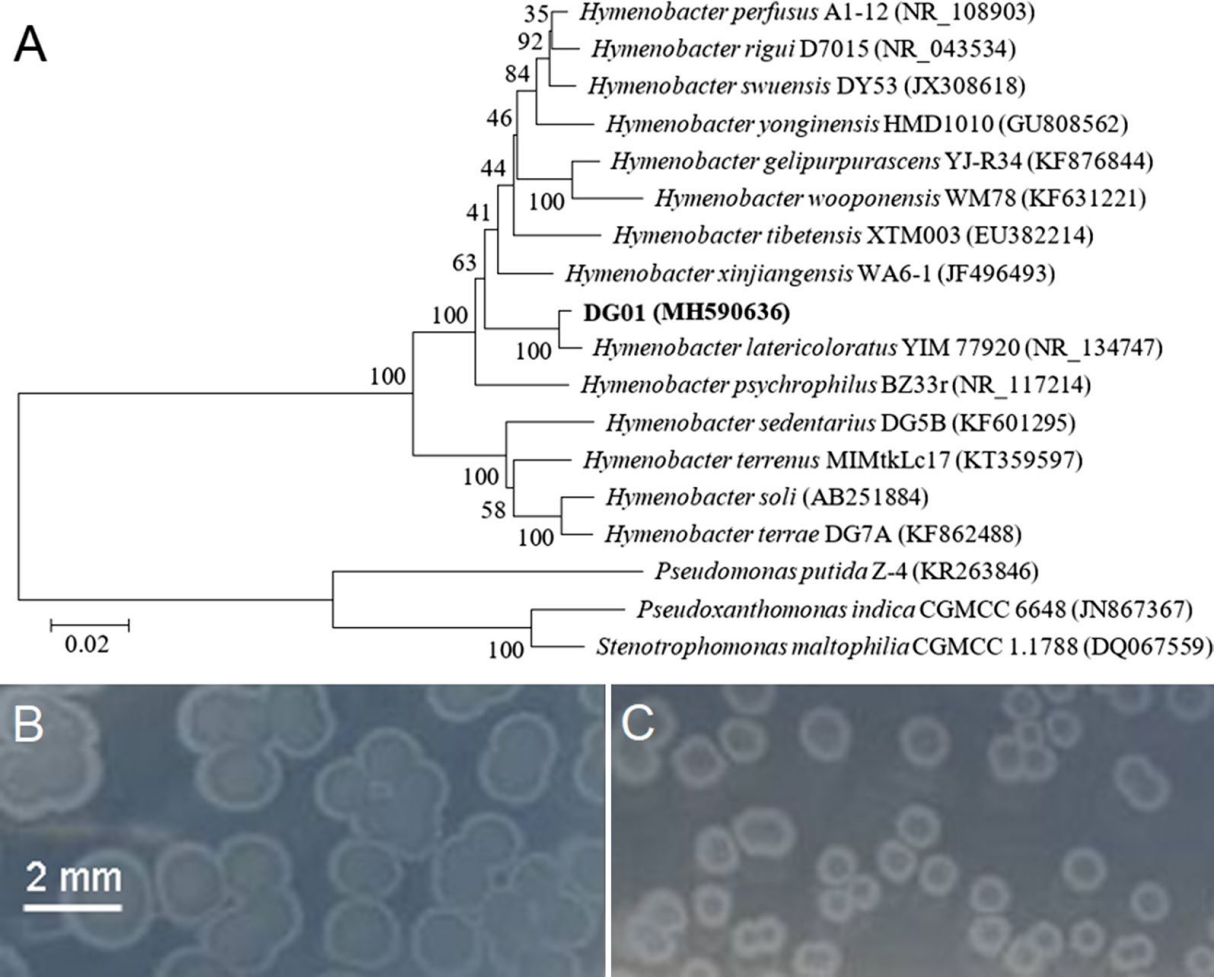
Neighbor-joining phylogenetic tree based on 16S rRNA gene sequences showing the phylogenetic relationship between strain DG01 and closely related taxa (**a**). The bar represents 0.02 substitutions per nucleotide position. Bootstrap values (expressed as percentages of 1000 replications) are shown at the branch points. The imidacloprid-degrading bacteria *Pseudoxanthomonas indica* CGMCC 6648 and *Stenotrophomonas maltophilia* CGMCC 1.1788 were used outgroups. Colonies of *Hymenobacter latericoloratus* CGMCC 16346 grown on 1/1000th (**b**) and 1/10000th lysogeny broth (LB) 2% agar plates (**c**). No bacterial colony was observed on the control plate containing agar only

Strain DG01 was Gram-negative, rod-shaped under light microscopy, and pink-pigmented on an R2A agar plate. Nucleotide BLAST and phylogenetic analyses of the 16S rRNA gene showed that DG01 clustered with *Hymenobacter latericoloratus* (Fig. 2a). *H. latericoloratus* strain DG01 was deposited in the China General Microbiological Culture Collection Center (CGMCC, Beijing, China) with accession number 16346.

### Confirmation that *H*. *latericoloratus* CGMCC 16346 is oligotrophic

Oligotrophic microorganisms including many bacteria and fungi grow in extremely nutritionally-deficient environments in which the concentrations of organic substances are low (Wyszkowska et al. 2016). As shown in Fig. 2b, c, *H*. *latericoloratus* CGMCC 16346 could grow on 1/1000th and 1/10,000th LB agar plates, indicating that it is an oligotrophic bacterium. However, *H*. *latericoloratus* CGMCC 16346 could not grow on full-strength LB agar plates, indicating that it is an obligate, not a facultative, oligotrophic bacterium.

### Metabolite identification on degradation of imidacloprid

**Fig. 3:**
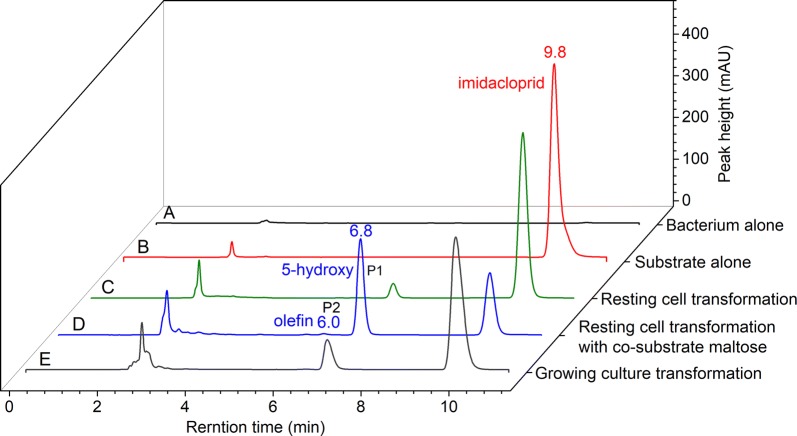
High-performance liquid chromatograms of imidacloprid degradation by resting cells and growing culture of *H. latericoloratus* CGMCC 16346. **a** Bacterial control without imidacloprid in resting cell transformation. **b** Substrate control without bacterial inoculation. **c** Resting cell transformation of imidacloprid by *H. latericoloratus* CGMCC 16346. **d** Resting cell transformation of imidacloprid by *H. latericoloratus* CGMCC 16346 in the presence of 2% maltose. **e** Transformation of imidacloprid by growing culture

**Fig. 4:**
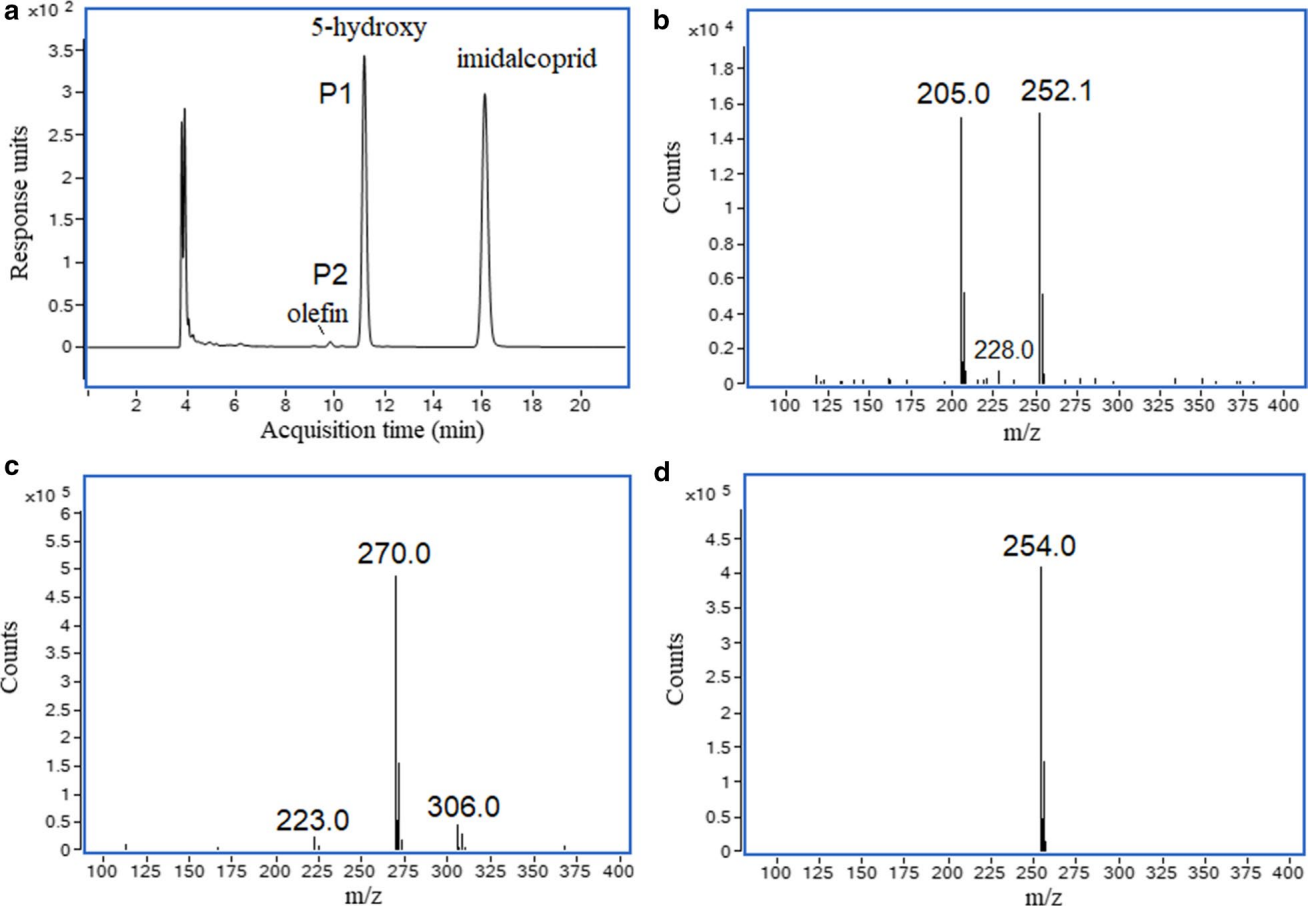
Liquid chromatography-mass spectrometry analysis of metabolites formed in the transformation of imidacloprid by resting cells of *H. latericoloratus* CGMCC 16346. **a** Chromatogram of resting cell degradation of imidacloprid. **b**–**d** Mass of the metabolites with retention times of 9.8, 11.2 and 16.0 min, respectively

As shown in Fig. 3, a metabolite P1, with retention time of 6.8 min, could be observed in the resting cell transformation of imidacloprid by *H*. *latericoloratus* CGMCC 16346. The bacterial control and substrate control did not produce this metabolite (Fig. 3a–c). When co-substrate maltose was added to the resting cell transformation broth, an additional minor metabolite, P2, was also observed with retention time 6.0 min (Figs. 3d, 4a). Figure 4 shows LC–MS analysis including an LC chromatogram (Fig. 4a), and mass spectra of P1, P2 (Fig. 4b, c), and the substrate imidacloprid (Fig. 4d). Metabolite P1 exhibited a parent ion [M−H]^−^ at *m*/*z* 270, an adduct fragment ion [M+Cl]^−^ at *m/z* 306, and an unknown fragment ion at *m/z* 223 (Fig. 4c). P2 exhibited a parent ion [M−H]^−^ at *m*/*z* 252, a tautomeric fragment ion [M−H−HNO_2_]^−^ at *m/z* 205, and a fragment ion of a protonated form [M+2H−H−C_2_H_2_]^−^ at *m/z* 228 (Fig. 4b) (Fusetto et al. 2016). Metabolites P1 and P2 had the same mass data and retention times as standard 5-hydroxy imidacloprid and olefin imidacloprid, respectively. Therefore, the metabolic pathway of imidacloprid degradation by *H. latericoloratus* CGMCC 16346 is via hydroxylation of imidacloprid to 5-hydroxy imidacloprid and the olefin imidacloprid metabolite (Fig. 1).

### Effect of co-substrate on imidacloprid degradation in resting cell transformation

A co-substrate, usually carbohydrate or organic acid, can be used as a source of energy and electron donor to enhance the degradation of organic contaminants (Lu et al. 2016). Glucose, maltose, pyruvate and succinate were respectively added into resting cell transformation broth to evaluate their effect on imidacloprid degradation by *H. latericoloratus* CGMCC 16346. As shown in Table 1, glucose, maltose and pyruvate increased imidacloprid degradation and 5-hydroxy imidacloprid formation. After transformation for 4 days, the imidacloprid degradation rate was 52.4%, 59.8% and 52.6% when using glucose, maltose and pyruvate as co-substrate respectively, whereas the control without co-substrate addition showed an imidacloprid degradation rate of only 9.8%. These results indicated that imidacloprid metabolism by pure culture of *H. latericoloratus* CGMCC 16346 involves a co-metabolism mechanism.

**Table 1:** Effect of co-substrate on the biodegradation of imidacloprid by resting cells of *H. latericoloratus* CGMCC 16346

Co-substrate	Content (mg/L)			Imidacloprid degradation rate (%)
	Reduced imidacloprid	5-Hydroxy	Olefin	
Glucose	51.6 ± 7.2a	36.9 ± 5.6a	1.4 ± 0.1a	52.4
Maltose	59.1 ± 11.4a	41.0 ± 10.3ac	1.6 ± 0.3a	59.8
Pyruvate	52.0 ± 15.6a	46.0 ± 13.10c	ND	52.6
Succinate	10.6 ± 2.1b	5.0 ± 1.7b	ND	11.6
Control	9.0 ± 3.9b	3.6 ± 0.6b	ND	9.8

The OD_600_ of the resting cell transformation broth was 5. The transformation time was 96 h. The data represent the mean values of triplicates. Mean values (± SD) within a column followed by different letters are significantly different at p ≤ 0.05 according to Duncan’s test

### Time course of degradation of imidacloprid by resting cells and growing culture of *H. latericoloratus* CGMCC 16346

**Fig. 5:**
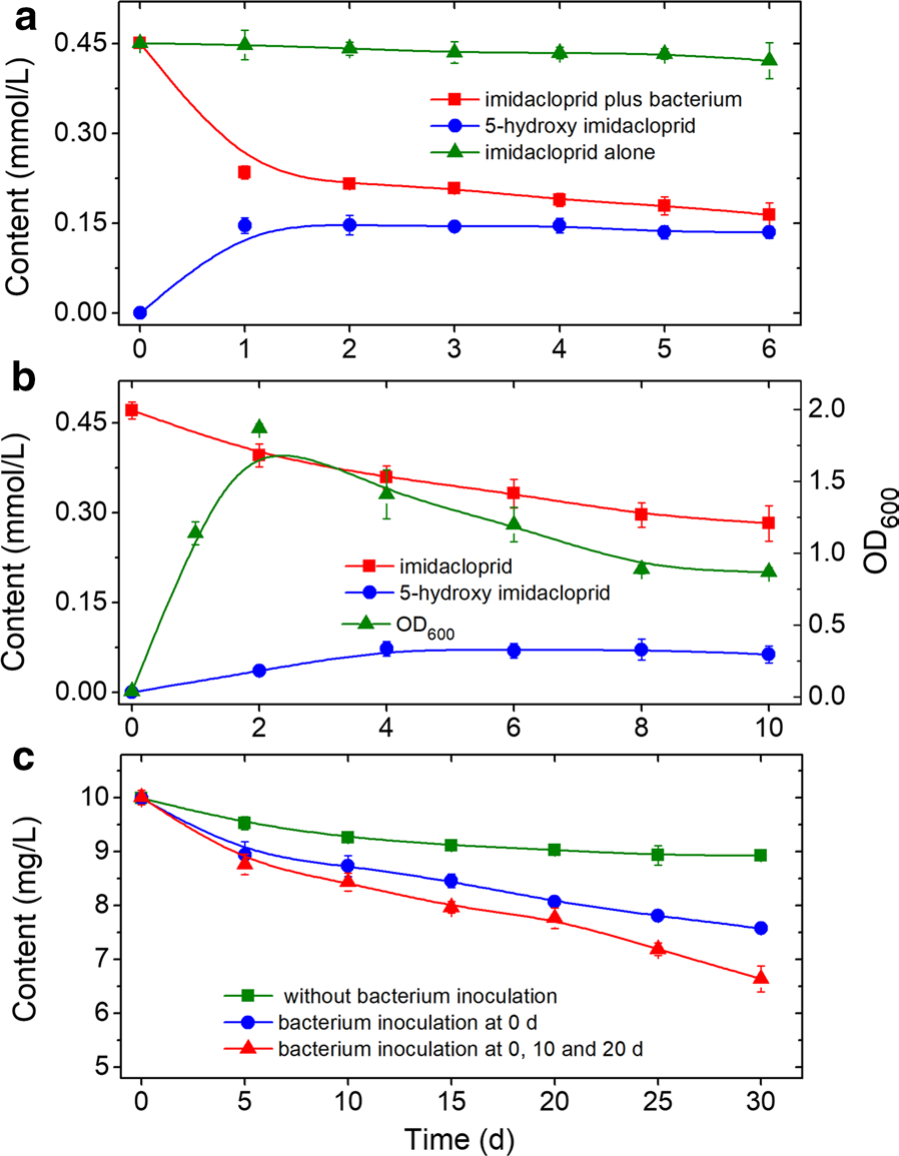
Time course of imidacloprid degradation by resting cells (**a**), growing culture (**b**), and imidacloprid remediation in tanks containing surface water (**c**). The resting cell transformation broth (**a**) contained 2% maltose as co-substrate and the OD_600_ = 5. The total volume of surface water (**c**) was 2.5 L inoculated with 3 × 10^7^ cells/mL

As Fig. 5a shows, resting cells of *H*. *latericoloratus* CGMCC 16346 degraded imidacloprid from the initial 0.45 mmol/L to 0.23 mmol/L in 1 day and the imidacloprid degradation rate was 48.9%. Meanwhile 0.15 mmol/L 5-hydroxy imidacloprid was formed. The molar conversion rate was 68.2%, indicating that hydroxylation was the main metabolic pathway of imidacloprid degradation. Subsequently, the imidacloprid degradation decreased, and only 19 μmol/L imidacloprid was degraded on the second day. After transformation for 6 days, the total amount of imidacloprid degradation was 0.29 mmol/L and the imidacloprid degradation rate was 64.4%.

As shown in Fig. 5b, imidacloprid could also be degraded in growing culture transformation. *H*. *latericoloratus* CGMCC 16346 degraded 40.1% of the imidacloprid (0.19 mmol/L) in 10 days, and the half-life of imidacloprid was 13.9 days. The 5-hydroxy imidacloprid formed peaked at 72 μmol/L on 4 days.

### Biodegradation of imidacloprid in surface water

The degradation of imidacloprid in surface water was primarily tested in 100-mL shaking flasks. As shown in Table 2, the control without bacterial inoculation showed slight imidacloprid degradation (5.3% in 10 days), while the imidacloprid degradation rate was improved to 16.8% by inoculation of *H*. *latericoloratus* CGMCC 16346. Addition of 1% co-substrate maltose did not enhance imidacloprid degradation compared with the control without maltose addition. These results indicated that *H*. *latericoloratus* CGMCC 16346 could degrade imidacloprid in surface water and co-substrate had no effect on the imidacloprid remediation.

**Table 2:** Degradation of imidacloprid in surface water in shaking flask system

Bacterial inoculation	Content (mg/L)		Degradation rate (%)
	Reduced imidacloprid	5-Hydroxy	
Control	0.56 ± 0.10a	ND	5.3
*H. latericoloratus* CGMCC 16346	1.79 ± 0.61b	ND	16.8
*P. indica* CGMCC 6648	0.22 ± 0.23a	ND	2.1
Maltose	0.40 ± 0.02a	ND	3.8
*H. latericoloratus* CGMCC 16346 + maltose	2.25 ± 0.85b	0.40 ± 0.12a	21.2
*P. indica* CGMCC 6648 + maltose	5.05 ± 1.94c	1.52 ± 0.25b	47.5

The cell inoculation amount was 2 × 10^8^ cells/mL. The maltose concentration was 1%. The imidacloprid content in water at day 0 was 10.6 mg/L. The data represent the mean values of triplicates. Mean values (± SD) within a column followed by different letters are significantly different at p ≤ 0.05 according to Duncan’s test

*ND* not detected

*Pseudoxanthomonas indica* CGMCC 6648 degrades imidacloprid via the same hydroxylation pathway as *H. latericoloratus* CGMCC 16346 (Ma et al. 2014). *P. indica* CGMCC 6648 could grow on nutrient broth and therefore is a copiotrophic bacterium. This bacterium was also tested for imidacloprid remediation in surface water (Table 2). Unlike the oligotrophic *H*. *latericoloratus* CGMCC 16346, inoculation with *P. indica* CGMCC 6648 did not increase the imidacloprid degradation rate compared with the control without bacterial inoculation in the absence of maltose. However, in the presence of 1% maltose, the imidacloprid degradation rate by *P. indica* CGMCC 6648 was improved to 47.5%. We previously showed that *P. indica* CGMCC 6648 has the same co-metabolism mechanism of imidacloprid degradation as *H*. *latericoloratus* CGMCC 16346 in pure culture, however, they show distinct differences in imidacloprid degradation in surface water. Although maltose can significantly enhance imidacloprid degradation by *P. indica* CGMCC 6648, the practical value of this is limited as maltose addition increased the chemical oxygen demand of the water.

In a 2.5-L tank, the imidacloprid content of the control without *H*. *latericoloratus* CGMCC 16346 inoculation was reduced from 10.0 to 8.9 mg/L after incubation for 30 days (Fig. 5c); the imidacloprid degradation rate was 11.0% and the half-life was 173.3 days (R^2^ = 0.86). After inoculation of *H*. *latericoloratus* CGMCC 16346, the content of imidacloprid at 30 d was decreased from 10.0 mg/L to 7.58 mg/L (Fig. 5c); the imidacloprid degradation rate was 24.2%, and the half-life was 86.6 d (R^2^ = 0.95). In experiments with batch inoculation of *H*. *latericoloratus* CGMCC 16346 on day 0, 10 and 20, imidacloprid was degraded from the initial 10.0 mg/L to 6.64 mg/L in d 30 (Fig. 5c); the imidacloprid degradation rate was 34.6% and the half-life was 57.8 days (R^2^ = 0.95). No metabolite 5-hydroxy imidacloprid was observed by HPLC. The above results indicate that *H*. *latericoloratus* CGMCC 16346 has the ability to remediate imidacloprid in surface water, and this imidacloprid-degradation activity remained after bacterial inoculation for 30 days. Furthermore, this imidacloprid degradation did not require supply of nutrients or co-substrate. We suggest that *H*. *latericoloratus* CGMCC 16346 is a potential bioremediation agent for elimination of imidacloprid contamination from water environments.

*Pseudoxanthomonas indica* CGMCC 6648 was also examined for imidacloprid degradation in surface water-containing tanks. *P. indica* CGMCC 6648 did not have the ability to degrade imidacloprid in these conditions. This strain showed the highest reported imidacloprid-degrading activity in pure culture, via the hydroxylation pathway (Ma et al. 2014). However, it lost the ability to degrade imidacloprid in surface water (oligotrophic conditions) and, therefore, cannot be used as a bioremediation agent for water environments polluted by imidacloprid.

### Genomes of *H. latericoloratus* CGMCC 16346 and *P. indica* CGMCC 6648

**Fig. 6:**
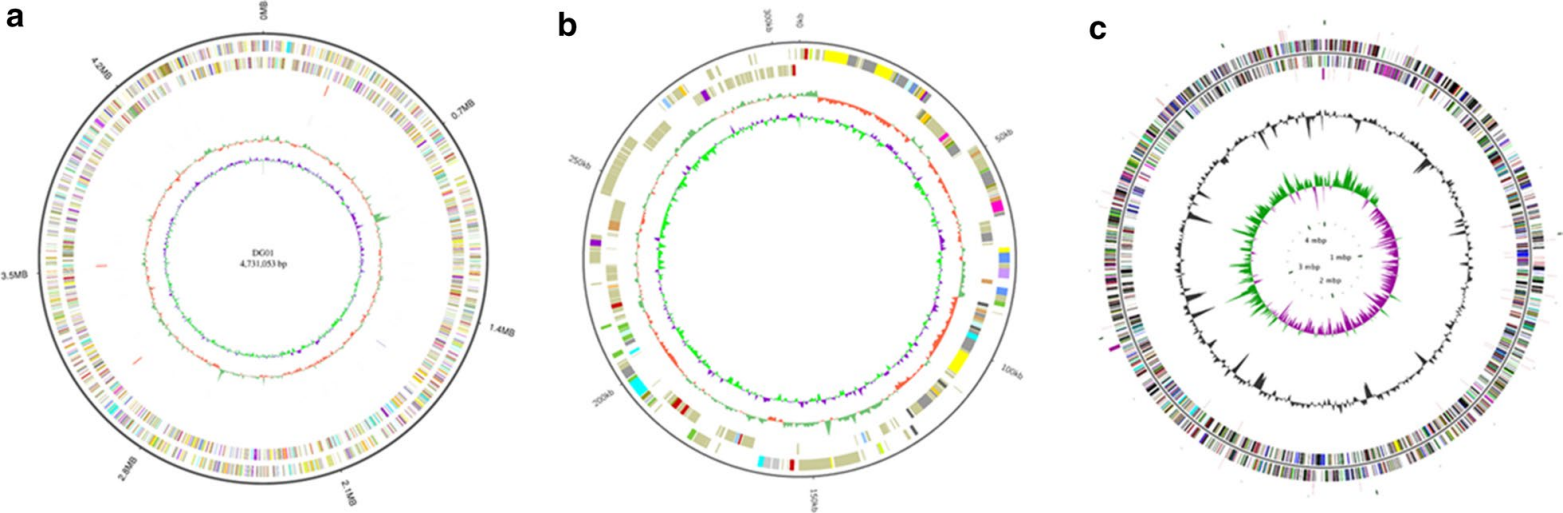
Genome maps of *H. latericoloratus* CGMCC 16346 and *P. indica* CGMCC 6648. **a** Chromosome of CGMCC 16346. **b** Plasmid of CGMCC 16346. **a**, **b** (from outer to inner): genome size, forward strand gene colored according to COG classification, reverse strand gene colored according to COG classification, forward strand ncRNA, reverse strand ncRNA, repeat, G + C content and GC skew. **c** Chromosome of CGMCC 6648 (from outer to inner): forward strand ncRNA, forward strand gene colored according to COG classification, reverse strand gene colored according to COG classification, reverse strand ncRNA, G + C content, and GC skew

The complete genome of *H. latericoloratus* CGMCC 16346 consists of 5,037,225 bp, with a chromosome of 4,731,053 bp and a plasmid of 306,172 bp. Maps of the *H. latericoloratus* CGMCC 16346 chromosome and plasmid are shown in Fig. 6a, b respectively. The chromosome of *H. latericoloratus* CGMCC 16346 contains 4133 predicted genes and the plasmid contains 291 predicted genes (total 4424 genes). *P. indica* CGMCC 6648 has a genome (chromosome) size of 4,304,170 bp (Fig. 6c) and 3935 predicted genes.

### COG comparison

COG categories were used to identify significant differences in imidacloprid degradation in surface water between the copiotrophic *P. indica* CGMCC 6648 and the oligotrophic *H. latericoloratus* CGMCC 16346. As shown in Table 3, The major COG categories in *H. latericoloratus* CGMCC 16346 were cell wall/membrane/envelope biogenesis (COG category M) (8.28%, percentage of all functionally-assigned genes); translation (J) (7.34%); amino acid metabolism and transport (E) (7.00%); and carbohydrate metabolism and transport (G) (6.80%). The major COG categories in *P. indica* CGMCC 6648 were amino acid metabolism and transport (8.29%); transcription (K) (7.50%); translation (6.42%); and energy production and conversion (C) (6.12%). A higher presence of COG category G (carbohydrate metabolism and transport; 6.80% in *H. latericoloratus* CGMCC 16346 vs. 5.37% in *P. indica* CGMCC 6648) reflects the relative availability of nutrients to these bacteria (Cobo-Simón and Tamames 2017). Hence, the oligotrophic *H. latericoloratus* CGMCC 16346 can grow on 1/10000th diluted nutrient medium, survive for a long time, and degrade imidacloprid in oligotrophic surface water, whereas *P. indica* CGMCC 6648 degrades imidacloprid in copiotrophic conditions with maltose supplementation (Table 2).

**Table 3:** Genomic features defining the lifestyle of oligotrophic *H. latericoloratus* CGMCC 16346 and copiotrophic *P. indica* CGMCC 6648

Marker		*H. latericoloratus* CGMCC 16346	*P. indica* CGMCC 6648
Genome size (bp)		5037,225	4,304,170
Total gene numbers		4424	3935
16S rRNA gene copy numbers		3	3
Total COG numbers		2970	2665
COG category			
B	Chromatin structure and dynamics	2	1
C	Energy production and conversion	156 (5.25%)	163 (6.12%)
D	Cell cycle control and mitosis	29 (0.98%)	28 (1.05%)
E	Amino acid metabolism and transport	208 (7.00%)	221 (8.29%)
F	Nucleotide metabolism and transport	63 (2.12%)	56 (2.10%)
G	Carbohydrate metabolism and transport	202 (6.80%)	143 (5.37%)
H	Coenzyme metabolism	155 (5.22%)	113 (4.24%)
I	Lipid metabolism	155 (5.22%)	109 (4.09%)
J	Translation	218 (7.34%)	171 (6.42%)
K	Transcription	163 (5.49%)	200 (7.50%)
L	Replication and repair	106 (3.57%)	108 (4.05%)
M	Cell wall/membrane/envelope biogenesis	246 (8.28%)	146 (5.48%)
N	Cell motility	29 (0.98%)	71 (2.66%)
O	Post-translational modification, protein turnover, chaperone functions	164 (5.52%)	126 (4.73%)
P	Inorganic ion transport and metabolism	161 (5.42%)	132 (4.95%)
Q	Secondary metabolites biosynthesis, transport and catabolism	86 (2.90%)	75 (2.81%)
R	General functional prediction only (typically, prediction of biochemical activity)	349 (11.75%)	336 (12.61%)
S	Function unknown	168 (5.66%)	227 (8.52%)
T	Signal transduction	155 (5.22%)	118 (4.43%)
U	Intracellular trafficking and secretion	30 (1.01%)	77 (2.89%)
V	Defense mechanisms	89 (3.00%)	43 (1.61%)
	Cytochrome P450	2	0

It is notable that COG category M (cell wall/membrane/envelope biogenesis) was better represented in *H. latericoloratus* CGMCC 16346 than *P. indica* CGMCC 6648 (8.28% vs. 5.48%). The genes involved in cell wall/membrane/envelope biogenesis strategies for creation of bacterial biofilms can promote colonization processes (Cobo-Simón and Tamames 2017). Consistently, COG category N, cell motility, is less well represented in *H. latericoloratus* CGMCC 16346 than in *P. indica* CGMCC 6648 (0.98% vs. 2.66%). This is in accordance with the fact that *Hymenobacter* is a non-motile bacterial genus and *Hymenobacter* species are often found in freshwater, potable water and household biofilms (Sun et al. 2018). In the present study, we observed that *H. latericoloratus* CGMCC 16346 easily adhered to the flask or tank wall containing surface water, whereas *P. indica* CGMCC 6648 did not. It is interesting that COG category V, defense mechanisms, is usually poorly represented in oligotrophic bacteria, but contained 3.0% of the genes in *H. latericoloratus* CGMCC 16346 compared with only 1.61% in *P. indica* CGMCC 6648. The enrichment of proteins for defense mechanisms enable *H. latericoloratus* CGMCC 16346 to resist attack by phage, which is quite frequent in water environments (Schmid et al. 2018).

### Analysis of cytochrome P450 monooxygenase genes and inhibition by PBO

**Fig. 7:**
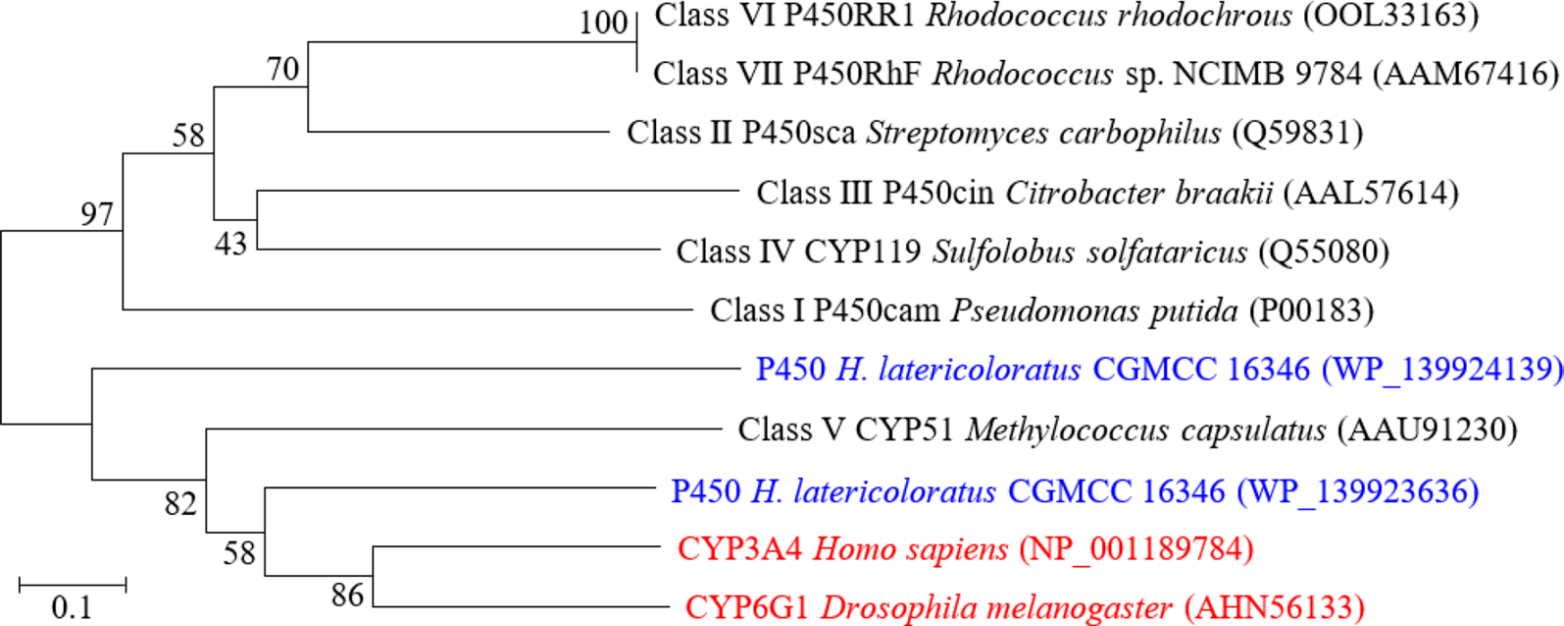
Neighbor-joining phylogenetic tree based on bacterial CYP450, human CYP3A4 and fruit fly CYP6G1 sequences. Human CYP3A4 and fruit fly CYP6G1 were reported to degrade imidacloprid via the hydroxylation pathway (Fusetto et al. 2017)

Human CYP3A4 and fruit fly CYP6G1 were proved to hydroxylate imidacloprid to its 5-hydroxy metabolite (Fusetto et al. 2017). Therefore, cytochrome P450 monooxygenases were searched in the annotated proteins of *H. latericoloratus* CGMCC 16346 and *P. indica* CGMCC 6648. There are two P450 monooxygenases in the chromosomal DNA of *H. latericoloratus* CGMCC 16346, with protein IDs WP_139923636 and WP_139924139 in GenBank, which are 467 and 445 amino acids long, respectively. Phylogenetic tree construction (Fig. 7) indicated that CGMCC 16346 P450 monooxygenase with accession number of WP_139923636 was clustered with human CYP3A4 and fruit fly CYP6G1. There is no P450 monooxygenase coding gene in the genome of *P. indica* CGMCC 6648, indicating that a monooxygenase other than a P450 enzyme is responsible for imidacloprid degradation through the hydroxylation pathway in that bacterium.

PBO is a specific inhibitor of cytochrome P450 monooxygenases (Wang et al. 2019); therefore, we examined the inhibition of imidacloprid degradation and hydroxylation by PBO. Imidacloprid degradation by CGMCC 16346 on PBO addition was 0.106 mmol/L, while that of the control was 0.153 mmol/L. The content of 5-hydroxy imidacloprid on PBO addition was 0.056 mmol/L, while that in the control was 0.122 mmol/L. PBO of 0.5 mmol/L thus inhibited 54.1% of 5-hydroxy imidacloprid formation. These results indicate that imidacloprid hydroxylation by *H*. *latericoloratus* CGMCC 16346 might involve the cytochrome P450 enzyme system. We respectively cloned the two P450 enzyme coding genes into pET28a and transformed the target plasmids into *Escherichia coli*, but the P450 enzymes could not be expressed. Overexpression of the P450 enzymes from *H*. *latericoloratus* CGMCC 16346 to prove their function in imidacloprid hydroxylation needs further study.

## Discussion

Imidacloprid has high water solubility (0.51 g/L at 20 °C) and persistence in soil, which have a high potential to run off into surface water and to leach into ground water (Sánchez-Bayo and Hyne 2014; Morrissey et al. 2015). Although imidacloprid was detected in surface water at level of micrograms per liter, it acts as an insect neurotoxin and is toxic even at low concentrations. Van Dijk et al. (2013) proved that imidacloprid concentrations as low as 0.01 ppb led to significant reduction in the number of macroinvertebrates in surface waters. Therefore, imidacloprid application in agriculture not only polluted water, but also produced risks to aquatic ecosystems. Here, we reported that the oligotrophic *H. latericoloratus* CGMCC 16346 isolated from water can remediate imidacloprid in surface water. *Hymenobacter* species have been isolated from a wide range of natural habitats (Sun et al. 2018). Type strains of *Hymenobacter* species have been found in a wide range of natural sources, including aqueous environments such as lakes, estuaries, coastal seawaters, glaciers, snow in Antarctica, and wetlands (Sun et al. 2018). For instance, *H. latericoloratus* YIM 77920 was isolated from freshwater sediment of Jiuxiang cave in Yiliang County, Yunnan Province, China (Liu et al. 2015). Recent metagenomic sequencing revealed that *Hymenobacter* was one of the dominant microorganisms in a partial nitrification biofilm and pink-pigmented household biofilms (Li et al. 2018; Xu et al. 2014).

Several microbes have the ability of degradation of imidacloprid in pure culture and they degraded imidacloprid via three pathways: hydroxylation to 5-hydroxy and olefin imidacloprid; nitroreduction to nitroso, guanidine and urea imidacloprid; oxidative cleavage to 6-chloronicotinic acid (Fig. 1). Among these imidacloprid-degrading microbes, *H. latericoloratus* CGMCC 16346, *S. maltophilia* CGMCC 1.1788 and *P. indica* CGMCC 6648 have the same hydroxylation pathway and co-metabolism mechanism in imidacloprid degradation by pure culture (Dai et al. 2007; Ma et al. 2014). However, *H. latericoloratus* CGMCC 16346 and *P. indica* CGMCC 6648 show significant differences in imidacloprid degradation in surface water. This phenomenon is related to the *H. latericoloratus* CGMCC 16346 is an oligotrophic bacterium isolated from water, whereas *P. indica* CGMCC 6648 is a copiotrophic bacterium isolated from soil. Our present studies focused on oligotrophic bacterium may provide a new strategy for screening neonicotinoid-degrading microbes and applying it for remediation in water environments.

The proteins of COG C (energy production and conversion), E (amino acid transport and metabolism), F (nucleotide transport and metabolism), G (carbohydrate transport and metabolism), H (coenzyme transport and metabolism), I (lipid transport and metabolism), P (inorganic ion transport and metabolism) and Q (secondary metabolites biosynthesis, transport, and catabolism) are responsible for the cellular metabolism (Cobo-Simón and Tamames 2017). The presence of COG G, H, I and P of *H. latericoloratus* CGMCC 16346 is higher than *P. indica* CGMCC 6648, while the presence of COG C and E of the former is lower than the later. We previously proved that co-substrates carbohydrate and organic acid enhanced the imidacloprid degradation was related to the metabolic flux of co-substrate metabolism through the glycolysis pathway, hexose monophosphate pathway and citric acid cycle, as well as cofactor NAD(P)H regeneration, not ATP regeneration (Liu et al. 2013). *H. latericoloratus* CGMCC 16346 has the higher presence of COG G and H results in it can efficiently utilize the low nutrient of the water under the oligotrophic conditions.

In conclusion, we found that *H*. *latericoloratus* CGMCC 16346 could degrade the globally-used neonicotinoid imidacloprid via hydroxylation. This bacterium remediated imidacloprid in surface water over a long period without addition of co-substrate. Comparison of genome features and COGs revealed that *H*. *latericoloratus* CGMCC 16346 is significantly enriched in the COG categories for cell wall/membrane/envelope biogenesis and defense mechanisms compared with *P. indica* CGMCC 6648. The present studies will aid understanding of the ecological function and lifestyle of oligotrophic *H*. *latericoloratus*, as well as helping development of *H*. *latericoloratus* CGMCC 16346 as an agent for imidacloprid bioremediation.

## Data Availability

The data supporting the conclusions of this article are included within the article. Data and materials can also be requested from the corresponding author.
